# Spinal scoliosis: insights into developmental mechanisms and animal models

**DOI:** 10.1007/s43390-024-00941-9

**Published:** 2024-08-20

**Authors:** Chongnan Yan, Guoxin Jin, Lei Li

**Affiliations:** Department of Spine Surgery, Shengjing Hospital of China Medical University, Shenyang, 110004 China

**Keywords:** Spinal scoliosis, Animal models, PINX, Zebrafish

## Abstract

Spinal scoliosis, a prevalent spinal deformity impacting both physical and mental well-being, has a significant genetic component, though the exact pathogenic mechanisms remain elusive. This review offers a comprehensive exploration of current research on embryonic spinal development, focusing on the genetic and biological intricacies governing axial elongation and straightening. Zebrafish, a vital model in developmental biology, takes a prominent role in understanding spinal scoliosis. Insights from zebrafish studies illustrate genetic and physiological aspects, including notochord development and cerebrospinal fluid dynamics, revealing the anomalies contributing to scoliosis. In this review, we acknowledge existing challenges, such as deciphering the unique dynamics of human spinal development, variations in physiological curvature, and disparities in cerebrospinal fluid circulation. Further, we emphasize the need for caution when extrapolating findings to humans and for future research to bridge current knowledge gaps. We hope that this review will be a beneficial frame of reference for the guidance of future studies on animal models and genetic research for spinal scoliosis.

## Introduction

Spinal scoliosis is a common spinal deformity that hinders the physical and mental state of affected individuals. Research indicates a crucial role of genetic factors in the occurrence of spinal scoliosis. However, the precise mechanisms underlying its pathogenesis remain unclear. In this review, we discuss current studies on the embryonic development of the spine and factors contributing to deformities, elucidating the genetic and biological mechanisms mediating axial elongation and straightening during the developmental process.

Zebrafish, due to its particular morphology, structure and genetic characters, is a widely employed model for investigations in developmental biology, genetics, and clinical medicine for a variety of conditions such as spinal scoliosis. We summarize the latest findings from studies on spinal scoliosis, with a particular focus on zebrafish, exploring the genetic and physiological processes of notochord development and cerebrospinal fluid dynamics. In addition, we discuss how defects in these processes contribute to the development of spinal scoliosis. Moreover, we have highlighted aspects that remain unknown as well as the potential application of novel animal models and genetic research that should be considered to guide future studies in understanding the pathogenesis of spinal scoliosis.

## Development of the spine (extension, straightening)

The developmental process of the spine is crucial for the normal growth of vertebrates. The notochord, originating from the mesoderm, exhibits a rod-like structural organization [1, 2]. During embryonic development, the notochord demonstrates diverse differentiation capabilities, giving rise to vital structures such as the neural tube, lungs, liver, pancreas, and gastrointestinal tract [3, 4]. Serving as a transient structure, the notochord gradually regresses and eventually disappears after embryonic development concludes [5].

During embryonic development, the vertebrate spine undergoes two key processes facilitating its elongation and straightening. First, notochordal cells undergo vacuolization. These cells, with a characteristic diameter of up to 50 μm, undergo continuous expansion, forming a bundle-like arrangement with fluid-filled interiors [6, 7]. The enlargement of these cell volumes establishes a statically stable framework capable of resisting mechanical stress, simultaneously maintaining the embryonic axis, and enabling the symmetric development of surrounding bone tissues [8, 9]. Moreover, these vacuolized cells undergo cavitation that divides the notochord into distinct functional differentiation units. Simultaneously, the surrounding sheath cell epithelium provides developmental guidance by inducing the migration of bone segments in the somite toward the midline and dorsal side. Ultimately, vertebral bodies and intervertebral fibrous rings form while muscle segments gradually construct the trunk muscles [10–12]. These two processes function in balance as some vacuolized notochordal cells accumulate below the ossification region and others fragment within the intervertebral disc tissue to form the nucleus pulpous [7, 13, 14].

In summary, the extension and straightening of the vertebrate embryonic axis involves interference with vacuolized notochordal cells and the sheath cell epithelium surrounding the notochord. Disturbances such as impaired vacuolization of notochordal cells, inadequate expansion of vacuolized cells, and fragmentation before maturation can lead to developmental abnormalities in the spine [8, 15, 16].

In addition, variations or deficiencies in the sheath cell epithelium and poor collagen secretion may render the notochordal sheath unable to effectively resist internal pressure, causing overexpression of ossification cells in certain regions, resulting in insufficient cartilage deposition, and ultimately triggering developmental abnormalities [17–19].

Spinal deformities in humans related to genetics typically include congenital scoliosis (CS), idiopathic scoliosis (IS), and neuromuscular or acquired scoliosis. Congenital scoliosis results from vertebral and rib malformations, occurring in approximately one in 1000 individuals [15]. Clinical and animal studies have shown that congenital scoliosis manifests as faulty vertebral segmentation, including hemivertebrae and wedge-shaped vertebrae [20, 21]. Current research indicates a strong correlation between congenital scoliosis and gene–environment interactions, with disruptions in the NOTCH signaling pathway playing a major role in vertebral segmentation loss under environmental stress [20, 22, 23].

In contrast, idiopathic scoliosis clinically presents as a three-dimensional rotational curvature of the spine without vertebral malformations, neurological-muscular abnormalities, or functional impairments. It affects about 4% of the population, representing over 80% of scoliosis cases, and is the most common spinal disorder in children and adolescents [24]. The term “idiopathic” signifies that the cause of this condition is not yet clear [25]. Several hypotheses related to the pathogenesis of idiopathic scoliosis have been proposed, including abnormalities in central nervous system development, abnormal spinal bone growth, bone metabolism, biomechanical environmental issues and hormonal imbalances [26–31]. Moreover, genetic factors play a crucial role as the probability of idiopathic scoliosis development is significantly higher in monozygotic twins compared to dizygotic twins (70% vs. 36%) [32]. In addition, there are genetic associations between congenital and idiopathic scoliosis [33–35].

**Fig. 1:**
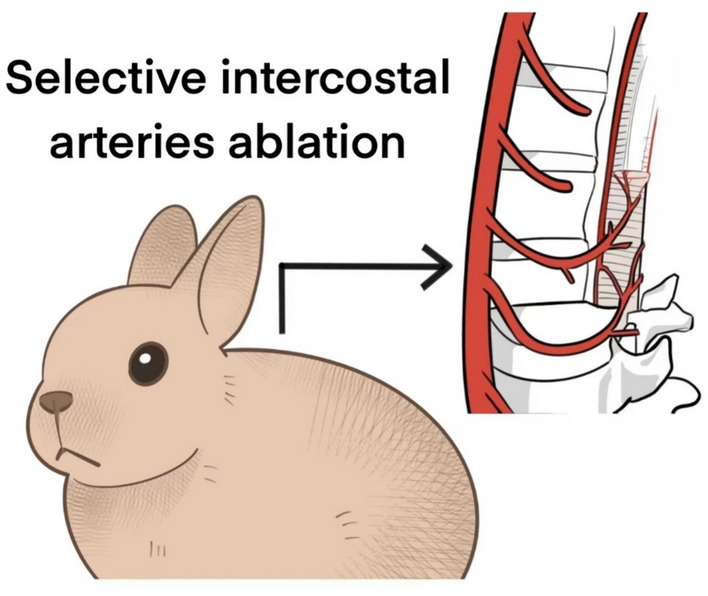
Selective intercostal arteries ablation

To study human spinal deformities, researchers have conducted extensive studies by establishing animal models of spinal deformities. Early research generally focused on natural observation of small animals, such as the progression of thoracic scoliosis in 5–6-week-old chicks. It was found that the imbalance of paraspinal muscle development causes scoliosis. More studies have shown that differential expression of collagen is an important factor in causing scoliosis [36]. In addition, researchers selectively ablated the intercostal arteries of the thoracic vertebrae at four levels in rabbits through thoracotomy to create a rabbit model of scoliosis (Fig. 1). During the experiment, it was observed that some animals developed scoliosis after selective intercostal artery ablation. Histological examination revealed changes in the vertebral epiphyseal plate and the spinal cord, affecting the proprioceptive function of paraspinal muscles in tone control [37].

**Fig. 2:**
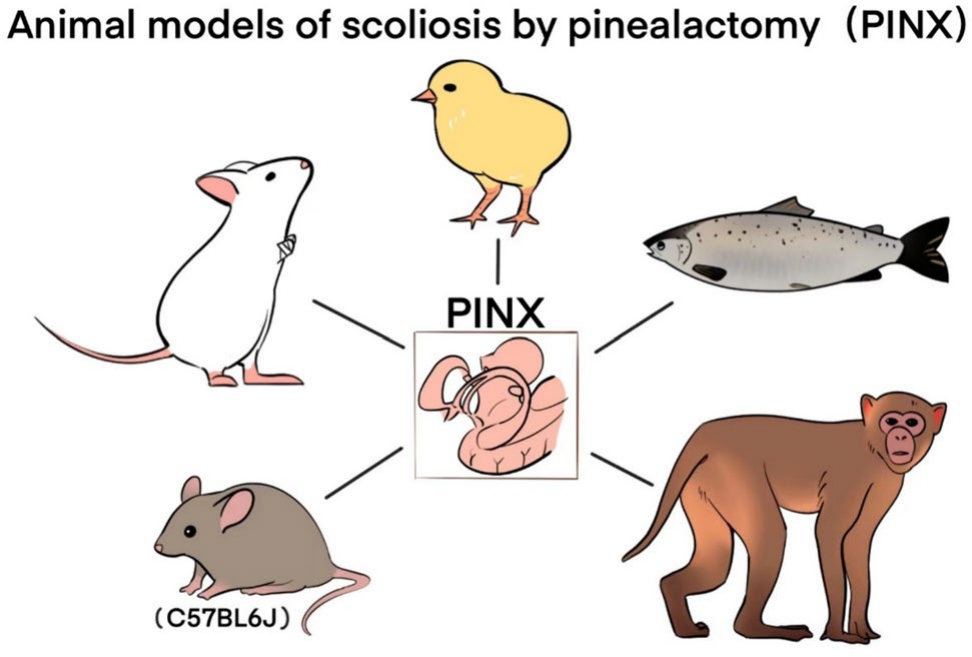
Animal models of scoliosis by pinealectomy

A series of studies have shown that melatonin plays a key role in the occurrence and development of scoliosis. Thus, a type of research in animal models uses pinealectomy (PINX) to create animal models of scoliosis (Fig. 2).

### PINX chicken model

In 1959, Thillard completed the first pineal gland excision (PINX) model. Postoperative spinal curvature was observed in 65% of pinealectomized chicken [38]. Until 1983, Machida and Duboussett extended this idiopathic scoliosis model through morphological correlation mapping [39]. It has been confirmed that the neurotransmitter or neurohormone system in the pineal gland is the main factor contributing to this experimental type of scoliosis.

To prove the hypothesis that melatonin deficiency is related to spinal deformity, Machida et al. concluded that melatonin metabolism plays an important role in the occurrence and development of scoliosis, with the total incidence rate of scoliosis in PINX chickens ranging from 80 to 100% [39–45]. However, other researchers have reported significant differences in scoliosis incidence [46–65]. Bagnall et al. used 105 newly hatched Shan Hubbard chickens. After 5 weeks of PINX melatonin treatment, over 50% of the chickens still suffered from scoliosis. Therefore, the use of melatonin alone cannot effectively reduce the incidence of scoliosis in PINX chickens.

### PINX fish (Atlantic salmon) model

To exclude the effects of body posture and gravity on spinal development and further demonstrate how melatonin deficiency alone promotes scoliosis, researchers evaluated the spinal development of PINX Atlantic salmon [66]. The spinal movement of salmon is mainly limited to lateral bending. The results of this study showed that 82% of pinealectomized fish exhibited abnormal spinal curvature. In addition, the calcium, phosphorus, and total mineral content in the vertebrae of PINX salmon were significantly reduced. It was concluded that melatonin may play a crucial role in spinal bone growth, bone mineralization, and the development of scoliosis.

### Congenital melatonin deficiency rodent (mouse) model and PINX bipedal rodent (rat) model

To further demonstrate the relationship between melatonin and induced scoliosis, researchers established a mouse model of AA-NAT gene knock-out strain (C57BL/6 J) with congenital melatonin deficiency. Many studies have found that the bone density (BMD) of C57BL/6 J mice is significantly lower, indicating that this model may be ideal for evaluating the effects of melatonin on bones [67–72]. Machida et al. used PINX bipedal C57BL/6 J mice to evaluate the development of scoliosis. The results showed that the spinal deformity rate of bipedal mice with melatonin deficiency was 25%. They believe that treatment with exogenous melatonin can prevent the development of scoliosis in both models. The same team achieved a higher induction rate of scoliosis by amputating the forelimbs of C57BL/6 J mice without PINX. The incidence of scoliosis in mice with simple forelimb amputation is twofold lower than in bipedal C57BL/6 J mice. However, when PINX was administered in melatonin-rich C3H/HeJ mice, the incidence of scoliosis increased to 70%. This result is consistent with early reports that spinal deformities are more likely to occur in the presence of both melatonin deficiency and bipedal posture.

Many researchers have conducted similar studies in rats. O’Kelly et al. conducted PINX in rats to evaluate the incidence of scoliosis [54]. Compared with chicken models, PINX alone cannot induce spinal curvature in any rodent model [39, 40, 42, 44]. Rodents that walk on four legs are different from vertebrates that walk on two legs and are not affected by the crucial dynamic mechanical force of posture leading to the development of scoliosis. Therefore, researchers developed a bipedal rat model (with two forelimbs and tails removed from 3-week-old rats, food and water placed at a high position to gradually enhance the upright posture). The conclusion is that in the melatonin deficiency model after pinealectomy, any interference with balance and other postural mechanisms, especially bipedal posture, may promote the development of spinal deformities.

### Non-human primate PINX (rhesus monkey) model

Due to the success of these experiments, researchers began to develop interest in non-human primates. In 2005, Cheung et al. conducted PINX in bipedal non-human primates [73]. Among 18 rhesus monkeys with PINX, 10 showed a significant decrease in melatonin secretion, but none of them showed the expected scoliosis after 29 months of postoperative follow-up. The limitation of this study is that monkeys are unable to move freely or stand upright in cages, which limits their natural posture [74]. Therefore, the spine is not affected by gravity, which is a crucial factor leading to the development of scoliosis. These findings further emphasize the importance of posture and posture-related mechanical forces, not just melatonin deficiency, which may be crucial for the development of scoliosis.

Recent studies have discovered spontaneous spinal deformities in wild populations of teleost fish, particularly zebrafish [75, 76]. Zebrafish are becoming increasingly popular among researchers for several reasons: (1) strong reproductive ability and large numbers; (2) similar spinal morphology and structure to humans, with many highly conserved genetic genes; (3) mechanical conditions of the spine during swimming in teleost fish are similar to human bipedalism; (4) transparent zebrafish embryos facilitate observation during external development; (5) comprehensive knowledge of the zebrafish genome allows for high-throughput bidirectional genetic regulation; and (6) the convenient size of the body for Micro-CT and scanning electron microscope analysis [77–86]. As a result, studies have been gravitating toward the use of zebrafish to establish models as well as to perform gene editing and sequencing to understand the pathogenesis of spinal deformities.

## Zebrafish model of scoliosis and related genes

**Fig. 3:**
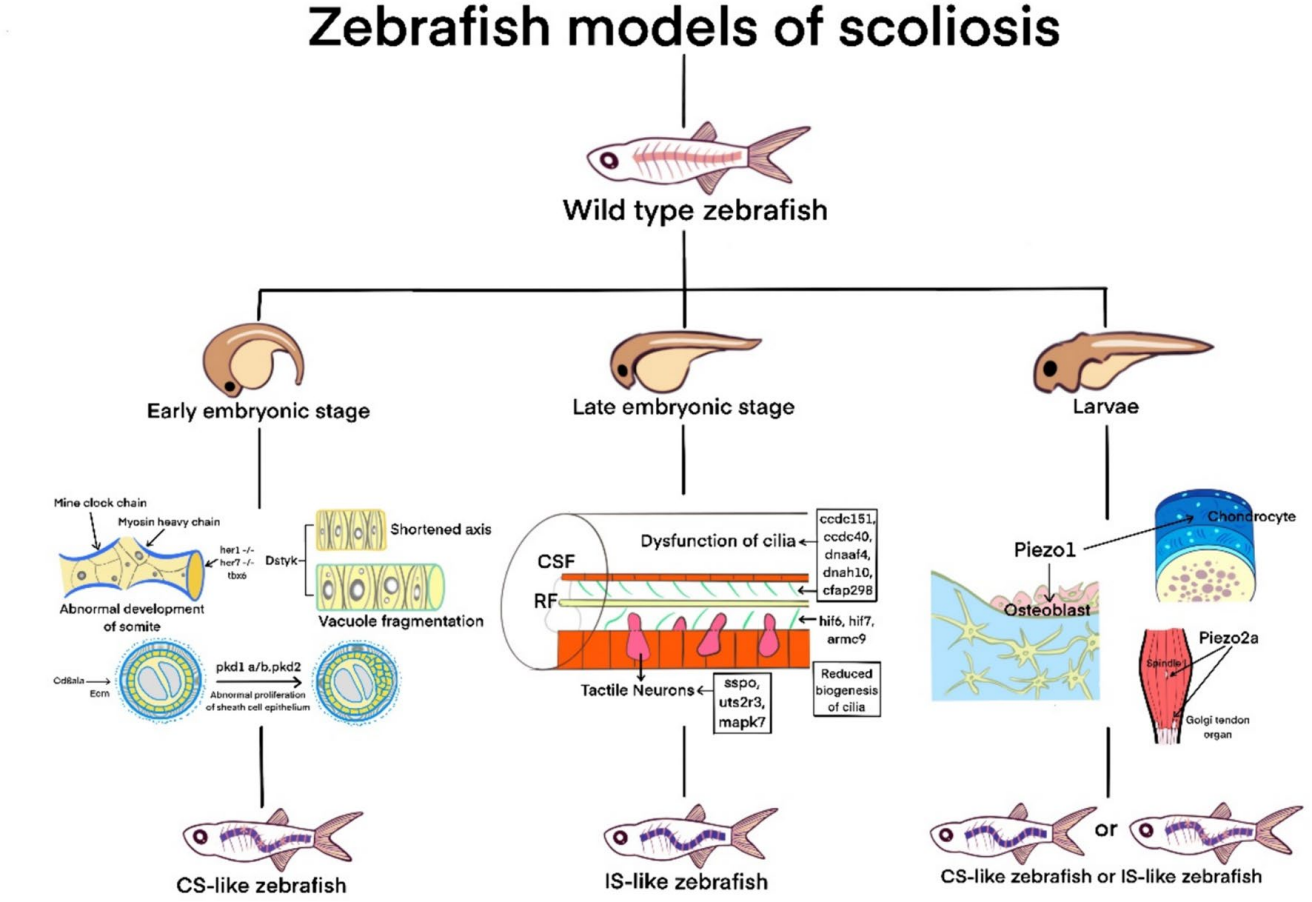
Zebrafish models of spinal scoliosis

Recent years, many researchers have intervened in the embryonic and juvenile development of zebrafish specimens through gene knock-in or knock-out methods, successfully producing zebrafish variants with spinal deformity (Fig. 3).

The establishment of a model of congenital scoliosis is generally achieved through gene editing and interference with the development of early embryonic notochord structures. It is reported that regulation of the activity of bisserine/threonine and tyrosine protein kinase (Dstyk) to reduce the number of vacuolized cells, shorten the embryonic axis, and thus affect spinal cord development, ultimately leading to late onset spinal deformities due to vertebral growth defects [87, 88]. Other ones overexpressed the pkd1a/b and pkd2 genes by upregulating them, leading to an abnormal increase in collagen in the epithelium of sheath cells of the notochord, resulting in excessive curvature of the dorsal side of the notochord and the formation of spinal deformities [89, 90]. The Leviathan variant is one of the more distinctive types. Mutations in the col8a1a gene result in the loss of type VIII collagen in the extracellular matrix of notochord cells, further affecting the position of osteoblasts and leading to vertebral defects in later development, resulting in spinal deformities [91]. In addition to interfering with the notochord, some zebrafish variants that interfere with the development and segmentation of somites to create congenital spinal deformities have also been reported. Mutations in genes such as mine clock gene, myosin heavy chain, tbx6, her1−/−, her7−/− can all lead to developmental disorders of early embryonic somites, resulting in spinal deformities [92, 93].

Compared with congenital spinal deformities, idiopathic spinal deformities have more complex causes and a lack of clear etiology. At present, most of the reported interference is achieved through the function of specific structures during late embryonic development and juvenile development. The maturation, activity, and function of motile cilia are one of the main targets for the preparation of idiopathic scoliosis models. By editing genes such as ccdc151, ccdc40, dnaaf4, dnah10, and cfap298, the activity of motile cilia is interfered with, causing zebrafish to gradually form idiopathic scoliosis during juvenile development [94, 95]. Regarding the biogenesis of motile cilia, some scholars have used genetic variations such as kif6, kif7, and armc9 to interfere and obtain variants of zebrafish with idiopathic spinal deformities [96–98]. Meanwhile, some reports have edited genes such as sspo, uts2r3, and mapk7 to interfere with Reissner fiber, vasopressin receptor, and bone formation processes, thereby inducing zebrafish specimens to develop into idiopathic scoliosis variants [99–102].

Although zebrafish are similar to humans in terms of spinal morphology and development, they also have differences. However, due to their outstanding advantages as experimental specimens and the rapid development of gene editing and sequencing technology, zebrafish provides us with new perspectives and favorable tools for in-depth biological research on scoliosis.

### The mechanical stress of the spine and the Piezo channel

After millions of years of evolution, humans have become bipedal vertebrates capable of upright walking, where the mechanical stress of gravity on the spine plays a crucial role. To maintain balance and achieve optimal range of motion in an upright posture, humans have evolved specific sagittal curvature of the spine and pelvis [103]. Importantly, it is the zebrafish that experience the same level of pressure exerted on the spine during swimming that recapitulate the effects of gravity on the human spine [76]. Recent studies have proposed sensory pathways capable of detecting mechanical stress and have identified the associated expression genes (Fig. 3). The Piezo pathway is widely present in the cell membranes of various human tissues, providing feedback for the conduction of mechanical stress. The Piezo genes include Piezo1 and Piezo2, with Piezo2 further divided into Piezo2a and Piezo2b [104]. Notably, Piezo1 gene is highly expressed in mesenchymal stem cells, osteoblasts, chondrocytes, and intervertebral disc nucleus pulposus [105]. Mutations or low expression of the Piezo1 gene can lead to reduced gene expression in chondrocytes and osteoblasts, resulting in wedge-shaped deformities of the vertebral bodies and thoracolumbar scoliosis [106–108]. Piezo2 gene plays a crucial role in proprioception and is mainly expressed in muscle spindles and tendon spindles. Mutations in this gene can cause joint deformities, vertebral fusion, spinal curvature, and hip joint malformation [109, 110]. When selectively knocking out the Piezo1 and Piezo2a genes in zebrafish, a model of spinal curvature is achievable in mature zebrafish [111]. The genes and pathways present in both the human body and teleost make the spinal deformity model more clinically meaningful. At the same time, the stage of action of this gene is more focused on the development direction of embryonic bone and cartilage, which is closer to the current understanding of the occurrence and development of spinal deformities. This achievement forms a robust foundation for future research in understanding the intricacies of spinal deformities.

## Cerebrospinal fluid circulation stability, motile cilia, Reissner fiber, and tactile neurons

The stability of cerebrospinal fluid (CSF) circulation is paramount for the homeostasis of the central nervous system [112]. In vertebrates, CSF secreted by the choroid plexus of the brain ventricles serves not only as a medium for nutrient transport and metabolic waste removal but also as a source of various signaling molecules [113]. Disruption of normal CSF circulation can lead to severe neurological disorders [114]. In zebrafish larvae, motile cilia are widely distributed in various tissues, including liver macrophages, olfactory cells, pronephric ducts, choroid plexus epithelium, and sperm [115, 116]. The rhythmic beating of motile cilia on the surface of the choroid plexus epithelium ensures the stable circulation of CSF in the brain ventricles and the central canal of the spinal cord [117–119]. Therefore, studies have found a close association between choroid plexus epithelial cilia dysfunction, reduced CSF flow, increased neuroinflammation, and spinal deformities in genetically edited zebrafish, suggesting that treatments or interventions targeting related factors can improve spinal deformities. However, such mutants often struggle to survive to adulthood which makes it a difficult model to study in the long term [120, 121].

In contrast, the circulation of CSF in humans primarily relies on arterial pulsation and respiratory movements. In individuals with primary ciliary dyskinesia, a condition where cilia are immotile, hydrocephalus is rarely observed, indicating that the function of motile cilia within the human spinal cord is not yet well understood. Further research is needed to comprehend the role of motile cilia in the development and homeostasis of the human ventricles [122–124].

Reissner fiber, initially described by Reissner in 1860 within the body of hagfish [125], is a highly conserved structure found widely in the spinal cords of vertebrates [126–130]. Although Reissner fiber was first observed in the human spinal cord in 1922 by Agduhr [131], subsequent studies using antibodies against glycoproteins specific to the connecting filum or Reissner fiber, derived from human or chimpanzee sources, failed to produce immune reactions [132–134]. As an extracellular glycoprotein, Reissner fiber extends linearly from the brain along the central canal of the spinal cord to its base [135]. In zebrafish, Reissner fiber aggregates in the cerebrospinal fluid within the brain ventricle through the secretion of the SCO-Spodin protein by the connecting filum, floating and traversing the central axis of the spinal cord [128]. Current research suggests that the absence of Reissner fiber does not affect ciliary movement and CSF flow, but the rhythmic beating of cilia plays a crucial role in the aggregation of Reissner fiber [128, 135]. However, the function of Reissner fiber remains unclear.

Tactile neurons (CSF-cNs) are GABAergic sensory neurons widely distributed on the ventral and dorsal sides of the central canal of the spinal cords in vertebrates [136]. Over a century ago, it was hypothesized that the connecting filum interacts with Reissner fiber to form a “sagittal position organ” with a mechanical sensory system that maintains the stability of the body axis [137]. Recent studies using zebrafish experiments have found direct contact between tactile neurons and Reissner fiber [138]. The absence of Reissner fiber renders tactile neurons incapable of responding to mechanical stimuli such as axial bending, indicating that the function of Reissner fiber is like that of a physical rope along the central axis, aiding tactile neurons in sensing changes in the body axis. Studies have conducted in-depth molecular studies on tactile neurons, indicating that the vasopressin neuropeptide expressed by tactile neurons, when activated by stimuli, causes the adjacent muscles to contract, maintaining the body axis and reducing bending. Abnormalities in the vasopressin signaling pathway can cause Tactile nerves (CSF-cNs) to be unable to sense the mechanical stress changes that occur in the Reissner fiber during trunk bending, making it difficult to adjust and control the axial development of the embryonic spine in time, ultimately resulting in spinal deformities [139–141].

## Considerations

Despite the significant contributions of numerous studies towards a deeper understanding of embryonic axial elongation, straightening, spinal development, and the molecular biology mechanisms underlying deformities, several critical considerations must be acknowledged:

1. The uniqueness of human spinal development: while extensive research has shed light on the molecular intricacies of axial elongation and spinal development in various vertebrates, the unique characteristics of human spinal development remain incompletely understood. As a bipedal species with distinct vertebral dynamics, the developmental processes in humans differ from those observed in other vertebrates [142, 143].
2. Physiological curvature discrepancies: the presence of natural physiological curvatures in the human spine (cervical lordosis, thoracic kyphosis, lumbar lordosis) contrasts with the generally straight spine observed in our closest relatives, such as chimpanzees [144].
3. Existing research results may not fully explain the mechanisms underlying the developmental changes in the human spine from embryonic to adult stages, including elongation, straightening, and physiological curvatures.
4. Cerebrospinal fluid circulation disparities: unlike experimental animals commonly used in research, human cerebrospinal fluid circulation relies significantly on arterial pulsation and respiratory movements. Consequently, the functional implications and potential effects highlighted by studies on motile cilia may not necessarily translate directly to spinal development and deformities in humans [133, 134].
5. Absence of tactile neurons and Reissner fiber in humans: the absence of conclusive evidence regarding tactile neurons and Reissner fiber (RF) in the human spinal cord raises serious doubts about the direct applicability of findings related to the interactions between motile cilia, tactile neurons, and RF observed in experimental animals [131–133].

In light of these considerations, it is imperative to approach the interpretation of research outcomes with caution, recognizing the unique aspects of human spinal development and the limitations in replicating experimental results directly to the human context. Future investigations are needed to address these specificities to advance our comprehension of the complexities associated with human spinal development and deformities.

## Summary and future perspective

### Critical role of the notochord in spinal development

The notochord emerges as a pivotal factor in the process of spinal development, playing essential roles in providing templates, organizing structures, and facilitating arrangements. The expansion, accumulation, and arrangement of notochordal vacuolated cells contribute significantly to the elongation of the spinal cord, maintaining axial alignment. However, the notochord’s involvement extends beyond spinal development, influencing the embryonic development of various vital organs. Precision and selectivity in intervening with upstream genes are crucial aspects to be addressed in future research, considering the potential impact on the overall development of embryos. Challenges arise, as interference may compromise the viability of experimental specimens up to maturity.

### Distinctive dynamics of cerebrospinal fluid circulation in humans

The unique modality of cerebrospinal fluid (CSF) circulation in humans sets it apart from other species. The mechanism underlying the role of motile cilia on the ependymal cells of the spinal canal’s membrane warrants further exploration. The distinctive nature of CSF circulation in humans demands an in-depth investigation into the functioning of motile cilia on the ependymal cells of the spinal canal’s membrane.

### Absence of evidence for Reissner fiber and tactile neurons in the human spinal cord

Currently, there is an absence of conclusive evidence supporting the existence of Reissner fiber and tactile neurons within the central canal of the human spinal cord. Consequently, caution must be exercised in extrapolating findings from animal experiments and genetic studies to the human context. As bipedal organisms, humans exhibit variations in spinal morphology throughout different growth stages. Understanding whether these variations correlate with microscopic structural changes in the spinal cord during fetal development, birth, and the transition to upright ambulation in adolescence is imperative. This evolution in spinal morphology in humans is likely influenced by a combination of external and intrinsic factors.

In summary, the intricacies of spinal development and related phenomena necessitate ongoing research that delves into the molecular and biomechanical intricacies. Rigorous exploration of these aspects will contribute not only to expanding our fundamental understanding of spinal deformities but also to the potential development of targeted interventions and therapeutic strategies in the future.

## Data Availability

Data availability is not applicable to this article as no new data were created or analyzed in this study.
